# Physical illness and multimorbidities in patients diagnosed with personality disorder

**DOI:** 10.1192/j.eurpsy.2024.212

**Published:** 2024-08-27

**Authors:** I. Simunovic Filipcic, N. Jaksic, S. Levaj, M. Sagud, I. Filipcic, M. Grah, D. Marcinko

**Affiliations:** ^1^Department of Psychiatry and Psychological Medicine, University Hospital Center Zagreb; ^2^University Psychiatric Clinic Sveti Ivan, Zagreb, Croatia

## Abstract

**Introduction:**

People with personality disorder (PD) often experience suffering, suboptimal psychiatric treatment outcomes, and early mortality due to chronic physical illness (CPI) and multimorbidity (≥2 CPI)  (CPM). Increasing research underscores the elevated prevalence of CPI and CPM in those with PD.

**Objectives:**

To compare the prevalence of CPI/CPM between the general population and those with PD and to explore the relationship between CPI/CPM and various aspects of PD.

**Methods:**

This cross-sectional study enrolled 126 PD patients (70.6% female, mean age 41.22 years) based on the ICD-10 criteria, and 126 socio-demographically matched individuals from the general population. The participants completed the following instruments: the ICD-11 Personality Disorder Severity Scale (PDS-ICD-11), the Personality Assessment Questionnaire for ICD-11 (PAQ-11), Subjective Emptiness Scale (SES), the Reflective Functioning Questionnaire-Revised-7 (RFQ-R-7), and self-reported chronic physical illnesses questionnaire.

**Results:**

The mean number of CPI in patients with PD and matched controls was 2.69 (SD=2.371) and 1.02 (SD=1.702), respectively, and this difference was statistically significant. Patients with PD also suffered more often from CPM than none or one CPI, compared to matched controls. In the multivariate logistic regression analyses among the patients with PD, higher personality disorder severity, increased trait Negative Affectivity and poorer reflective functioning/mentalizing were predictive of having CPM. These relationships were independent of age, gender, education status, income level, length of psychiatric treatment, and smoking status. Subjective emptiness was not significantly predictive of having CPM.

**Conclusions:**

This study highlights a higher prevalence of CPI and CPM in individuals with PD compared to the general population. Factors such as higher PD severity, increased Negative Affectivity, and poorer reflective functioning were identified as predictors of CPM. These findings underscore the necessity for integrated healthcare approaches to address the multifaceted needs of PD patients, emphasizing the importance of considering both mental and physical health in treatment strategies.

**Disclosure of Interest:**

None Declared

